# An Evidence Roadmap for Implementation of Integrated Behavioral Health under the Affordable Care Act

**DOI:** 10.3934/publichealth.2015.4.691

**Published:** 2015-10-20

**Authors:** Bethany M. Kwan, Aimee B. Valeras, Shandra Brown Levey, Donald E. Nease, Mary E. Talen

**Affiliations:** 1 Department of Family Medicine, University of Colorado School of Medicine, Aurora, CO, United States,; 2 NH Dartmouth Family Medicine Residency, Concord Hospital Family Health Center, Concord, NH, United States,; 3 Northwestern Family Medicine Residency, Northwestern McGaw Medical Center and University, Chicago, IL, United States

**Keywords:** Integrated behavioral health, primary care, affordable care act, collaborative care, depression, implementation science

## Abstract

The Affordable Care Act (ACA) created incentives and opportunities to redesign health care to better address mental and behavioral health needs. The integration of behavioral health and primary care is increasingly viewed as an answer to address such needs, and it is advisable that evidence-based models and interventions be implemented whenever possible with fidelity. At the same time, there are few evidence-based models, especially beyond depression and anxiety, and thus further research and evaluation is needed. Resources being allocated to adoption of models of integrated behavioral health care (IBHC) should include quality improvement, evaluation, and translational research efforts using mixed methodology to enhance the evidence base for IBHC in the context of health care reform. This paper covers six key aspects of the evidence for IBHC, consistent with mental and behavioral health elements of the ACA related to infrastructure, payments, and workforce. The evidence for major IBHC models is summarized, as well as evidence for targeted populations and conditions, education and training, information technology, implementation, and cost and sustainability.

## 1. Introduction

The health care system in the United States has been re-oriented to promoting quality patient-centered care at a lower cost for the entire population [1]. Such an approach requires attention to heretofore-marginalized mental and behavioral health concerns, which historically had been largely managed separately from physical health, or not at all. The Patient Protection and Affordable Care Act (ACA) of 2010 presented many opportunities and incentives for expanding access to and sustainability of behavioral health services in the U.S.[2]. As discussed by Croft and Parish [2], the ACA presents pathways to increased adoption of integrated behavioral health care (IBHC) by 1) enhanced access to care through increased insurance coverage (e.g., through Medicaid expansion and health insurance exchanges) and mental health parity (i.e., mental health and addiction services must be covered as essential benefits on the exchanges), 2) finance and reimbursement changes that support mental and behavioral health care through increased Medicaid and Medicare payments for primary care, health homes for those with serious mental illness, accountable care organizations (ACOs), co-location of primary care and behavioral health in community-based behavioral health settings, and home and community-based support services for Medicaid beneficiaries; and 3) infrastructure supports, including funds for workforce development and training programs. Thus, the ACA supports a number of structural, financial and workforce development changes needed to adopt and sustain high quality IBHC.

IBHC (also sometimes referred to as collaborative care [3]) is broadly defined as a set of elements (structures and processes) designed to address a range of mental and behavioral health needs in concert with primary care (Table 1). Dissemination of IBHC is increasing, but evidence-based models and interventions are often not implemented with fidelity, and there is wide variability in how IBHC is defined and implemented in real world settings [4],[5]. At the same time, there are only a few truly evidence-based models, especially beyond depression and anxiety, and further research and evaluation is needed. Given the ACA's emphasis on implementation and dissemination of evidence-based interventions [6], those seeking to implement IBHC would benefit from knowing the evidence. The purpose of this paper is therefore to support health care organizations (from small, independent practices to large fully integrated health care systems) seeking to implement IBHC by presenting an evidence roadmap, to inform decisions about the structures and processes needed to do integrated care well.

**Table 1.** Integrated Care Elements and Definitions

Elements of Integrated Care	Definition
Care delivery team	Patients & family, provider, nurse, care managers, pharmacists, and Behavioral Health Clinicians (social workers, psychologists, psychiatrists, therapists)
Education, training and practice preparation	Establishing buy-in and stakeholder engagement in planning; workforce development, training programs, continuing education, in-services, conferences, informal consultation, team-building exercises
Information Technology	Access to shared computers, telephones, electronic medical records, email, registries, dashboards and portals for tracking outcomes, telemedicine and mobile health technology, access to data for Quality Improvement (QI)
Setting	Whether in a free-standing clinic, or part of hospital system, dedicated physical or virtual space for BHC to interact with providers, teams, and patients.
Targeted populations and conditions	Universal services vs. prioritizing patients of a certain age (children, adults, elderly); level of risk, or with certain types of conditions (depression, anxiety, serious mental illness) or psychosocial concerns
Clinical processes	Screening and population identification protocols, risk stratification algorithms for appropriate level of care, access, treatment, monitoring and referral protocols
Cost / Sustainability	Securing funding (fund-raising, grants, advocacy, partnerships with payers), appropriate allocation of resources, receipt of payment for billable services
Office management policies and protocols	Established leadership and development of practice mission and values, time and effort protocols, privacy policies, billing and coding protocols, incentives and support for collaboration, and QI policies

## 2. Roadmap to the evidence

**Integrated behavioral health care is effective**

A driving factor in the decision to pursue organizational change – and IBHC is a major change – is evidence for the effectiveness of a new approach to care. Numerous reports on the evidence for IBHC conclude that the evidence supports the use of integrated care for managing both depression and anxiety in primary care settings, while noting that the effectiveness varies across settings, populations, and targeted health concerns [7]–[10]. In 2008, Butler and colleagues published an in-depth report on mental health integration in primary care, concluding that the purported benefits of integrated care for managing both depression and anxiety were supported by the evidence [7]. Several years later, Butler produced another report showing that integrated care improves depression outcomes, but noted level of integration (e.g. degree of shared treatment decision making or extent of co-location) in the care process or in provider roles was not related to better outcomes [8].

In their systematic review, Oxman, Dietrich, and Schulberg [11] described the research on collaborative care models as representing a third generation of research on the treatment of depression in primary care, following a first generation of multifaceted, collaborative care interventions and a second generation grounded in the principles of the chronic care model and guideline-based care. In this third generation, there was increased emphasis on effectiveness rather than efficacy in the context of translation, dissemination and sustainability, and attention to aging populations. An enhancement of “consultation-liaison skills” and better relationships between primary care clinicians and mental health specialists was considered an important advancement in the field. While Oxman et al concluded that referral to specialty mental health care would likely lead to better outcomes at an individual level, they also acknowledged that overall population health would be best improved with the more limited care made available from within primary care because of increased access.

Consistent with Oxman et al [11], Gilbody and colleagues' meta-analysis revealed considerable heterogeneity in effects for earlier studies (in the 1980s and 1990s), while the post-2000 evidence demonstrated more stable estimates of the effectiveness of IBHC for managing depression [12]. Gilbody and colleagues also found that the degree of effectiveness was related to medication adherence and the professional background and supervision method of case managers, specifically the use of case managers with mental health training and regular, planned supervision. More recently, Thota and colleagues again concluded that collaborative care for depression is effective at both the individual patient and public health levels [10], and can be economically viable [13]. In another review, Katon and Selig [14] noted that a population-based approach that coordinates the care of depression from within primary care should be particularly effective for reducing overall prevalence of depression. They suggest that three activities well-suited to primary care are key to secondary prevention of depression – improved diagnosis (including screening for risk factors and early evidence of minor depression), preventing chronicity, and preventing relapse/recurrence by virtue of more frequent contact and opportunities for tracking and monitoring symptomology.

Thus, IBHC is, overall, known to be effective for increasing access to behavioral health services and improving outcomes, with the evidence primarily supporting effectiveness in the domain of depression. As discussed below, much of the research to date has focused on depression and thus the body of evidence for conditions beyond depression remains small. Once acknowledging the value of and deciding to adopt IBHC, the next decision to be made concerns HOW to adopt an IBHC approach. An understanding of the evidence for classic (e.g., care management for depression) and contemporary (collaborative care systems addressing a range of behavioral health needs [15]) models and frameworks can inform the HOW, depending upon the needs, resources, and priorities of the organization.

## 3. Models and Frameworks

Systematic reviews of the evidence for IBHC have examined several of the IBHC models [7],[10],[16],[17]. A brief sampling of the models subjected to research and formal evaluation are described here and summarized in Table 2. Each model is characterized in terms of key elements, including the care team, the setting, the consultation and referral arrangements, and the clinical processes (including screening, triage, treatment, and monitoring treatment response) (see Table 1 for definitions of these elements).

***Care management for depression.*** In general, the model with the most empirical support for its effectiveness is care management for depression (i.e., IMPACT) [7],[8]. IMPACT was originally conceptualized as a chronic disease management program for older adults with depression [18],[19]. IMPACT involves a team-based approach to managing depression from within primary care. The acute and maintenance phases of depression are tracked by the care manager, a nurse or psychologist who provides education, care management, and medication support or brief psychotherapy.

In the initial grant-supported, multi-site randomized trial, those in the intervention group had higher rates of depression treatment (odds ratio [OR] = 2.98 [2.34, 3.79], p < .001) and experienced significantly greater odds of 50 percent reduction in depression symptoms than those in the usual care group (OR = 3.45 [2.71, 4.38], p < .001) [19]. Usual care patients were also screened for depression, and could receive treatment for depression through existing channels. Evidence also suggested that the intervention led to lower healthcare costs over a four-year period [20]. More than fifty publications have resulted from research on IMPACT, with overall favorable results.

Similarly, the Prevention of Suicide in Primary Care Elderly: Collaborative Trial (PROSPECT) study utilized care managers who used a protocol-based intervention to monitor depression treatment adherence and response and provide guidelines-based recommendations to physicians, who were the sole decision makers [21]. Compared to usual care, the intervention led to increased access to depression care, greater declines in suicidal ideation, earlier treatment response, and higher rates of remission at 4, 8 and 24 months [22],[23].

***Three component model.*** The three component model (TCM) is also characterized by care management and enhanced mental health support, and explicitly includes the concept of a “prepared practice” [24]. A prepared practice is one in which providers have received education on how to follow new practice protocols [24]. The Re-Engineering Systems for Primary Care Treatment of Depression (RESPECT-D) project was a cluster randomized trial of an intervention based on the three-component model [25]. Intervention patients had approximately double the odds of achieving a 50 percent reduction in depression symptoms as well as remission at 3 and 6 months. The project was supported by training manuals and quality improvement resources, rather than research protocols and grant funding – potentially making this a more sustainable approach [26]. The implementation and evaluation of RESPECT-D in the military setting (RESPECT-Mil) for the treatment of service members with post-traumatic stress disorder and depression showed that the three component model was feasible, acceptable, and led to clinically significant improvement in that context [27].

***Co-located collaborative care.*** The Strosahl [28] primary mental health care model of co-located collaborative care is distinguishable from the aforementioned care management models because master's or doctoral level mental health specialists are located onsite in a primary care clinic and provide services to patients of that clinic, often in collaboration with a PCP.

While widely adopted as a collaborative care model, there is limited empirical evidence on this model, with a few exceptions. In the Primary Care Research in Substance Abuse and Mental Health for the Elderly (PRISM-E) study, co-located mental health and primary care for mental health/substance abuse was compared to enhanced referral to specialty mental health care [29]. In PRISM-E, there was evidence demonstrating that co-located collaborative care led to increased access to mental health and substance abuse services compared to enhanced referral [30]. However, clinical outcomes were generally comparable across the two conditions, although enhanced referral to specialty mental health appeared to be superior for patients with major depression [31],[32].

The US Veterans Health Administration has embraced collaborative care, and has implemented a variety of models, including care management models targeted to depression [33] and other mental health conditions [34], and a blended model (co-location plus care management) in a number of their practices across the country [35]. The VA's White River Model incorporates comprehensive mental and behavioral health care into primary care, with co-located behavioral health providers (therapists and psychiatrists) as part of the care team, information technology to support assessment and tracking, care management, and chronic disease management. Screening and triage are also important processes of care. Patients can receive brief or long-term individual psychotherapy or group psychotherapy for a number of mental disorders, including depression, anxiety, stress/anger management, post-traumatic stress disorder, and substance use. Based on “before-after” study designs, this model appears to have led to improvements in access to care, patient and provider satisfaction, and adherence to evidence-based guidelines for depression treatment, and decreased cost of mental health care in the context of this capitated, single payer system [36],[37]. Furthermore, in a comparison with VA facilities that had not implemented this model, facilities with mental health integration showed greater increases in rates of detection of mental health disorders [38].

***Primary care in specialty mental health.*** Sometimes referred to as “reverse integration”, primary health care can be provided to patients with severe mental illness in specialty mental health settings, either through co-located primary care providers or care coordination. The VA system has also tested several reverse integration models [39]–[41]. For instance, the Primary Care Access, Referral, and Evaluation (PCARE) study is a randomized trial of primary care management for patients with severe mental illness being cared for in a community mental health center [39]. At the PCARE 12-month follow-up, intervention patients were significantly more likely than usual care patients to have received recommended preventive services (58.7% vs 21.8%), to have experienced greater improvements in mental health status, based on the SF-36 (8% improvement vs 1% decline), and to have lower cardiovascular risk, based on Framingham Cardiovascular Risk scores [39].

**Table 2.** Components of major published IBHC models and frameworks

Model	Care team	Setting	Consultation and referral	Clinical processes
**Care management for depression (IMPACT)**	Depression care manager (nurse, social worker, psychologist) Primary care provider Consulting psychiatrist	On-site primary care (care manager) Remote (psychiatrist)	Psychiatric consultation considered if clinically indicated Care plans are discussed with the PCP and the consulting psychiatrist	Routine screening for depression Stepped care approach to managing depression, with a 3-step evidence-based treatment algorithm Treatment options include antidepressant medication, brief psychotherapy Regular telephone follow up for a year (weekly at first, and then less frequent as depression lessens)
**Three-component model**	Care manager Clinicians Psychiatrist	Care management centralized in an organization or localized within a practice	Psychiatrist supervises and provides guidelines for the care manager, provides consultation services to the PCP, and facilitates appropriate use of additional mental health resources	Care management: patient education, counseling for self-management and adherence, assessment of treatment response and communication with other clinicians Spectrum of services through telephone calls and limited psychotherapy Psychiatrist prepares a practice to implement the model through initial and ongoing psychiatric education re: diagnosis, risk assessment and care plans
**Co-located collaborative care model**	Mental health specialists (masters or doctoral-level psychotherapists) Primary care providers	Mental health services provided within primary care	As needed	Co-located mental health specialists provide traditional psychotherapy (e.g., cognitive behavioral therapy) as well as “curbside” consultation for PCPs Triage: in which level of care is increased depending on patient need, risk or severity, ranging from behavioral health consultation, to specialty consultation, to fully collaborative care Appropriate training and re-training of expectations, for both mental health and medical care providers
**Primary care in specialty mental health**	Mental health specialty providers Nurse care managers Primary care providers	Community mental health centers	As needed	Nurse care managers encourage patients to seek medical care for their medical conditions through patient education and motivational interviewing and assist patients with accessing and navigating the primary care system through advocacy and addressing system-level barriers, such as lack of insurance

## 4. “Active ingredients” of IBHC?

Past attempts have been made to determine “active ingredients” of IBHC – those structures and processes necessary and sufficient for effective IBHC. In a review from the Canadian Collaborative Mental Health Initiative (CCMHI), Craven and Bland [16] reached conclusions supporting several elements of integrated care as key factors in improving outcomes, including practice preparation, co-location, collaboration (especially when paired with treatment guidelines), systematic follow-up, patient education, sensitivity to patient preference, and counseling to promote treatment engagement and adherence. In a meta-analysis and meta-regression of specific intervention content, eight aspects of these interventions that varied across 34 studies on collaborative care for depression were tested as predictors of depression outcomes [12]. These variables included setting (USA vs non-USA), recruitment method, patient population, PCP training, case manager background, case management sessions, case manager supervision, and case management content. Of these, four were at least marginal predictors of depression symptoms in multivariate analyses—setting (in favor of non-USA studies), recruitment method (in favor of systematic identification through screening rather than referral by clinicians), care manager background (in favor of those with mental health expertise), and care manager supervision (in favor of those receiving regular/planned supervision). While difficult to separate from other aspects of multifaceted interventions, care management does appear to be an important factor in depression care [17]. However, care management functions in different ways across different contexts, and it is not clear which are the most effective components, which background or training is needed, or whether ongoing supervision of care managers is necessary.

The extent to which members of the care team collaborate (versus provide separate care in parallel) distinguishes many practices that provide integrated care. In a meta-analysis of studies evaluating the effects of interactive communication between primary care clinicians and specialists - defined as “direct, personal interaction with specialists… such as curbside consultations” (Foy, p. 247) [42]—randomized trials involving collaboration between PCPs and psychiatrists exhibited a small to medium effect size for mental health outcomes in favor of collaboration. This is consistent with recent findings of a Congressional Budget Office review of Medicare Demonstration Projects, which found that in-person interactions between care managers, providers and patients were uniquely associated with programs that demonstrated improved outcomes [43]. This in-person interaction can be contrasted with enhanced referral and/or collaboration from afar. While some have concluded that referral to specialty mental health care would likely lead to better outcomes at an individual level, it was also acknowledged that overall population health would be best improved with the more limited care made available from within primary care because of increased access [11].

## 5. Targeted Populations and Conditions

Decisions about care teams, care delivery settings and clinical processes to be implemented may also be influenced by the characteristics of the patient population served. While comprehensive services for all may be ideal, practical limitations dictate the need to narrow down the population to be targeted by IBHC services, and the types of clinical concerns to be managed internally versus referred out. The skills and training of the care team, needs of the patient population, available resources—and the evidence—inform the decision about targeted populations and conditions.

***Targeted Populations.*** The evidence base for IBHC addresses certain populations more than others, including older and middle age adults, veterans, and patients cared for in HMO settings, although the evidence is still limited to the disease contexts previously noted. For instance, both IMPACT and PROSPECT focused primarily on geriatric populations. In contrast, there is a limited amount of evidence on integrated care for children and adolescents. The Youth Partners-in-Care (YPIC) study was an RCT of the effects of a care management quality improvement intervention compared to enhanced usual care, in youth ages 13 to 21 with depression [44]. Although generally consistent with standard care management, YPIC care managers were masters or doctoral level psychotherapists who delivered cognitive behavioral therapy (CBT) or coordinated delivery of other treatment options, and were not supervised by additional mental health specialists. Modest but statistically significant improvements in depression outcomes and patient satisfaction were observed. Some limited evidence exists for IBHC for peripartum women [45] and ethnic minorities such as Hispanic/Latino(a) patients [46].

***Targeted Conditions.*** The main body of evidence in IBHC concerns the management of depression, a pervasive and burdensome illness but by no means the only mental health problem confronted in primary care. Growing evidence exists in other mental health domains, such as panic disorder [47], substance abuse and addiction [32],[48], and bipolar disorder [49]. In the Netherlands, a RCT comparing collaborative stepped care versus care as usual for the treatment of panic disorder and generalized anxiety disorder in primary care showed improvements in the group receiving the collaborative stepped care model, which held one year post−test [difference in gain scores from baseline to 3 months: −5.11, 95% confidence interval (CI) −8.28 to −1.94; 6 months: −4.65, 95% CI −7.93 to −1.38; 9 months: −5.67, 95% CI −8.97 to −2.36; 12 months: −6.84, 95% CI −10.13 to −3.55] [50],[51]. This approach was applied to patients with other mental health diagnoses, including depression, and the group receiving the collaborative stepped care model experienced an earlier treatment response, at four months post-test, compared to care as usual (74.7% v. 50.8%; p = 0.003), but no significant differences between the groups eight and 12 months post-test as both groups showed improvement [52]. The VHA has tested several telemedicine models for patients with post-traumatic stress disorder (PTSD). RESPECT-PTSD was based on the three-component model (telephone care management, with psychiatrist supervision), which did not improve symptoms or functioning but did increase use of mental health services [53], and the total number of care manager calls was positively correlated with number of psychiatry visits (*r* = 0.63, *p* < 0.05) and amount of reduction in PTSD symptoms (*r* = 0.66, *p* < 0.05) [54]. In the VHA's TOP study (also primarily telemedicine), the off-site care team included nurse care managers, pharmacists, psychologists and psychiatrists; using interactive video technology, psychologists provided cognitive processing therapy, and psychiatrists conducted psychiatric consultations. This study did show significant improvements in PSTD symptoms among the intervention arm patients [55].

Much of the most recent literature on IBHC involves management of multiple psychiatric and/or physical comorbidities. Many IBHC model features (e.g., care management, interdisciplinary collaboration, clinical monitoring and follow-up, stepped care) reflect an instantiation of Wagner's Chronic Care Model [56], and thus can be used to co-manage multiple chronic diseases. It is also thought that treating mental illness may have direct and/or indirect effects on other illnesses, possibly because of physiological, social, cognitive, and/or behavioral factors common to the comorbid conditions [57]. In a pilot study of a patient-centered depression care management intervention characterized by several elements of integrated care (e.g., education and adherence monitoring), elderly adults with comorbid depression and hypertension were found to have lower depression scores, lower blood pressure, and greater medication adherence at six weeks [58].

Based on the IMPACT model, the Multifaceted Diabetes and Depression Program (MDDP) targets comorbid diabetes and depression in a low-income, predominantly Hispanic population [59]. MDDP incorporates several IMPACT-like features, with diabetes depression clinical specialists (DDCSs) serving in the care manager capacity, stepped care for depression, supervision by a PCP, and an available consultant psychiatrist. In addition, MDDP involved “sociocultural enhancements” (e.g., addressing social stigma towards mental health), education and counseling in self-management of both depression and diabetes, and patient navigation services. Consistent with the results of other combined depression-and-diabetes collaborative care interventions [60] and subgroup analyses of patients with diabetes in the original IMPACT study [61], MDDP resulted in improved depression, functioning and financial status and reduced symptom burden for both depression and diabetes – but there were no objective effects on diabetes control (e.g., change in HgA1c).

There are mixed results regarding whether effective treatment of mental illness (in the context of IBHC) can lead to improved outcomes for comorbid chronic diseases. Longer term follow-up and/or the addition of more intensive chronic disease-specific intervention content may be required to observe an effect on these other outcomes. For instance, the Stepped Care for Affective Disorders and Musculoskeletal Pain (SCAMP) study implemented a twelve-week antidepressant therapy intervention in sequence with a six session pain management intervention (followed by a six month continuation phase) in patients with comorbid depression and musculoskeletal pain [62]. Treatment algorithms were coordinated by nurse care managers in primary care settings, who were supervised by a physician depression specialist. Not only did patients in the intervention experience significantly greater improvements in depression than those in usual care, they also experienced significantly greater improvements in pain severity and interference.

The TEAMCare intervention focused on patients with diabetes or coronary heart disease or hyperlipidemia and depression and utilized nurse case managers with specialist consultation working with primary care physicians in an attempt to increase adherence to medication and other self-care behaviors for both depression and co-morbid physical illnesses [63]. The TEAMCare intervention failed to demonstrate significant effects on medication adherence, but led to significant changes in provider prescribing behavior [64]. An early implication of these findings is that treating mental illness may aid in improving coping skills (e.g., emotion coping) and self-regulation/self-management, which have subsequent salutatory effects on stress and pain, which helps to improve functioning and quality of life – even if short term effects on medical illnesses are not observed.

A broader focus on the range of behavioral health needs of patients in primary care [15], including basic psychosocial needs, health behavior modification, and the myriad mental health conditions presenting in primary care is much less common in the research literature. There is evidence demonstrating the effectiveness of behavioral medicine interventions in primary care settings [65],[66] and limited but compelling literature on how to integrate behavioral medicine in primary care [67].When broadly focused models are evaluated, the designs are generally less rigorous, the outcomes studied are generally more process-oriented (rather than clinical), and the conclusions are less generalizable outside the context in which the evaluation took place. The primary exception to this rule is that reverse integration models often seek general medical care (e.g., not just for diabetes) for a range of patients cared for in specialty mental health (e.g., not just patients with schizophrenia). By design, necessity and/or default, these broad health focused models are concerned with process and system capacity, such as defining and expanding the roles of health care professionals (e.g., advanced practice nurses) [68].

## 6. Workforce Development, Education and Training

A critical component of IBHC is the workforce needed to deliver behavioral health services in collaboration with primary care. However, there is a looming workforce shortage of mental health professionals who can deliver quality behavioral services in primary care settings [69]. Behavioral health providers from case managers to master's level therapists to doctoral level or medical providers are not well equipped to practice at the top of their license in these settings [69],[70]. Furthermore, the vast majority of educators and supervisors of behavioral health and mental health field have not practiced or developed skills in IBHC settings. Opportunities for training and education in IBHC are in embryonic stages of development and it is rare for mental health providers to have participated in coursework, supervised clinical experience or direct experience in primary care settings. The ACA recognizes the workforce shortage and supports education and training in mental health and behavioral health professions. The ACA's Mental and Behavioral Health Education and Training Grants (Sec. 5306 MBHETG of the ACA) are expected to influence the future training of behavioral health providers. Consequently, the ACA supports “programs designed to increase the number of professionals and paraprofessionals (to) service high priority populations ….and plan to service medically underserved in health professional shortage areas or in medically underserviced areas.” [71]. Recommendations for workforce development include incentive programs that recruit providers to underserved settings, like the National Health Services Corps, which could support integrated training clinical training programs [72].

***IBHC competencies and curriculum.*** In order to prepare for a new breed of mental health providers to work in primary care settings, there needs to be a foundation of knowledge, skills and professional values that support this training. However, mental health training has lagged behind other healthcare professions in defining core competencies for behavioral healthcare professionals. The Interprofessional Education Collaborative is one interdisciplinary organization whose goal is promote new models of team-based care and interprofessional communication for healthcare reform [73]. Behavioral health groups such as social workers, counselors, psychologists, psychiatric nurses, and psychiatrists are beginning to re-tool their professional competencies toward IBHC training [74]. There are a number of guidelines from psychologists, psychiatric nurse practitioners, social workers and psychiatrists that outline a set of core competencies for integrating their professions into primary care [75]–[78].

Creating team-based care training, clinical experience in primary care and quality improvement focus are essential for new models of care [69],[79],[80]. The Annapolis coalition on Behavioral Health Workforce Education [74] outlined the recommendation for improving the relevance of graduate education. They proposed several areas for improvements; some of the key elements include competency based training using evidence-based practice guidelines along with values, knowledge and skills for new models of care—including population management and interdisciplinary approaches. Essentially, educators are proposing a paradigm shift from a narrow focus on individual mental health to four pillars of IBHC practice: 1) evidence-based practices of integrated care, 2) research methods, 3) interprofessionalism and 4) quality indicators and outcomes [71]. Training in integrated care requires a broad range of services at a faster pace, of shorter duration, and with frequent team-based communication [70]. The roles of behavioral health in ACA organizations, therefore, are focused on expanding traditional mental health skills and services. These models include implementing BH into the medical home through establishing workflows for complex patients, expanding the workforce through clinical training programs, strengthening evidence for IBHC and designing evaluation protocols to assess impact of IBHC [81].

***Current state*** and ***future needs in training and education.*** Even though there is an urgent call for training a new breed of mental health professionals, we lack data on the effective training models, adequate credentials, and team configurations [72]. Models of training or practice that may be valid in mental health specialty clinics may not be applicable for primary care settings. The specific roles, tasks, and skills for diverse mental health providers in primary care are not well understood. While there is a growing number of within discipline training sites, certificate programs, or re-tooling programs that offer IBH in primary care, there is no consistency or definition on what constitutes the basics of integrated primary care training [69]. Consequently, education and training need to be evaluated and developed for the growing IBH workforce from a range of disciplines.

Table 3 outlines the disciplines, training, clinical experience and evidence-based practices that are currently available. At this stage, there is an urgent need for all mental health disciplines to evaluate our current training models that contribute to quality IBHC practice.

**Table 3.** Supported disciplines and training for IBHC

Discipline and Degrees	Academic Training	Clinical Experience and Practice level	Evidence-Based Practice	Re-Tooling
Associates Bachelors	Case management, Social Work	Screening, supportive counseling, referral and coordination of care	Chronic mental health (MH) management (IMPACT)	---
Master's level (Social Work, Counselors, Family Therapists) LCSW, LPCC, MFT	Competency-based curriculum (professional guidelines) Team-Based Care	MH screening, Warm hand-offs; patient education, EBP psychotherapy, Substance Abuse	Chronic MH management (IMPACT, IAPT) Brief CBT	Certification programs (e.g. University of Massachusetts, University of Michigan)
Doctoral level (PsyD, PhD) Psychology/Social Work, Behavioral Primary Care)	Competency-based curriculum (APA guidelines); Research Methods Quality Indicators Team-Based Care	MH screening, Warm-hand offs, assessment and diagnosis; EBP psychotherapy; QI and research initiatives, Team-based care, population health	Chronic MH management for depression, anxiety, diabetes (IMPACT, IAPT) Brief CBT	Certification programs; Internships (VA, DoD, APA sites) , post-doctoral fellowships
Medical (Nurse practitioners, Psychiatrist, Primary Care Physician)	Competency -based	MH assessment and diagnosis; EBP and consultation; Team-based approach	Chronic MH management (IMPACT, IAPT)	AIMS; select residency rotations; SAMHSA
Other: Interprofessional Team-Based Care	Competency-based	Team communication; values, QI, process roles and tasks		IPEC

Note: AIMS = APA = American Psychological Association, CBT = cognitive behavioral therapy, DoD = Department of Defense (United States), EBP = Evidence-based practice, IAPT = Improving Access to Psychological Therapies (United Kingdom), IPEC = Interprofessional Education Collaborative, SAMHSA = Substance Abuse and Mental Health Services Administration (United States),

## 7. Health Information Technology

Another important structural component of IBHC is the health information technology (HIT) used to ensure care is “patient-centered, evidence-based, measurement-based, population-based, and accountable” [82]. Specific HIT features that can assist in the aforementioned improvements to care include tracking and monitoring of patients for registry development, management, and appropriate outreach, and administering appropriately timed screenings that include skip logic to limit patient survey burden. HIT for IBHC can be as basic as having an established telephone number at which care managers, on-site behavioral health providers, and off-site consultants can be reached. Not as easily accommodated is the need for behavioral health templates in the electronic health record (EHR) and the opportunity for primary care providers and behavioral health providers to share care plans. Shared care plans are a core feature of truly collaborative care, although innovative strategies are often needed [83].

Notably, HIT supports clinical decision support (CDS) with evidence based practice treatment algorithms. Intermountain Healthcare in Utah developed a model of mental health integration that combines evidence-based treatment with innovative informatics tools (e.g., electronic health records, registries, electronic clinical decision support) for tracking patient progress and navigation of the system [84]. The goal was to enhance detection, monitoring, and management of mental health conditions, enhance patient and family engagement to support adherence and self-management, and treatment matching and adjustment. These tools support a model of risk stratification, in which progressively more intensive treatment is provided as risk level increases or persists, with universal screenings for and continued diagnostic assessment of those at risk [85].

HIT can assist with better patient-centered care with greater focus on prevention while working to increase patient engagement and health literacy [86]. Patients can access their medical records and use secure patient portals to communicate securely with providers, and contribute information to their record—part of “meaningful use” of EHRs [87]. Furthermore, mobile health (mHealth) applications can support complex self-management through myriad mechanisms including alerts, symptom monitoring and feedback, and assistance with barriers to adherence [88]. Some EHRs are now linking with mobile apps and other third party systems to better integrate health data, such as Duke University and Ochsner Health System in Louisiana integrating with Apple HealthKit via Epic [89]. One study supported patients' receptivity to using mobile devices as approximately one-half of patients surveyed predicted that mHealth would improve the convenience, cost and quality of their healthcare in the next three years, while six in ten doctors and payers believe that its widespread adoption is inevitable [90]. In this study, many also reported that though they believe mHealth will eventually become an important part of care provision; they expect that adoption will take time due to multiple barriers. Technology-enabled delivery of behavioral interventions may also increase the accessibility of evidence-based practices by increasing patient acceptance and/or extending the workforce capacity to deliver such interventions [91],[92].

Many HIT-facilitated functions in IBHC, such as registry management and hospital follow-up performed by care managers or social workers, fall within the domain of care coordination. At present, many practices use homegrown care coordination systems separate from EHRs as reporting tools to accomplish the aforementioned tasks, sometimes in time-consuming ways, as current EHRs often do not offer features in a way that the clinics need to coordinate care [93],[94]. Because of this, EHRs today need further development of features that the patient-centered medical homes require to improve efficiency, quality, and safety [94]. Additionally, practices need continued financial and technical assistance for enhanced HIT adoption [95]. Further HIT development is needed in areas such as monitoring registry functions, notifying teams when specific patients move across care settings, developing tools that enable team-based and patient centered IBHC, enhancing reporting activities, improving clinical decision support, and cultivating interoperability for more effective care delivery [93],[94].

***Telemedicine.*** Telemedicine deserves special attention in a discussion of HIT for IBHC. Several telemedicine models have been studied [96],[97]. These models include antidepressant consultation with an off-site psychiatrist via video conference [98],[99], telephone-based care management for depression in patients recovering from coronary artery bypass graft [96], telephone care management plus cognitive behavioral psychotherapy for patients taking antidepressant medication [97],[100],[101]. The use of telemedicine for delivering mental health services has been popular in rural Australia [102], predominantly for assessment and consultation rather than psychotherapy, with trends over time showing increased access to care.

The VHA tested the TEAM (Telemedicine Enhanced Antidepressant Management) intervention [98], which consisted of annual screening for depression using the PHQ-9, and a depression care team that provided a stepped-care model of depression treatment to patients screening positive for depression. This model was essentially a variation on the theme of IMPACT, but with telepsychiatry rather than on-site psychiatry, using interactive video technology. The team was comprised of an on-site PCP, a consulting psychiatrist available via teleconference, and off-site nurse depression care managers, clinical pharmacists and supervising psychiatrists. The stepped care treatment included 1) watchful waiting or treatment with antidepressant medication (ADM), with symptom monitoring by the care manager; 2) given non-response to the initial ADM, the psychiatrist, PCP, and clinical pharmacist consulted (generally via a note in the EHR) to make further recommendations; 3) given further non-response, a telepsychiatry consultation was recommended; 4) a final step was referral to specialty mental health at the parent VA medical center. Usual care patients were also screened for depression, had their depression scores entered in to the EHR, and had interactive video equipment available at the point of care for specialty mental health consultation. The results of this randomized trial demonstrated no difference in rate of prescription of ADM, but the intervention led to significantly higher odds of 50 percent improvement in depression severity at 6 months, and of remitting at 12 months [99]. This rural telemedicine collaborative care intervention was, however, more expensive than its urban, on-site counterparts [103]. As already described above, the VHA has also had success with a telemedicine-based collaborative care intervention for PTSD [55].

## 8. Implementation of IBHC

Once having decided on structures, processes and targeted populations and conditions, and staffed your model with well-trained clinicians, the next critical step to be guided by the evidence is implementation. Research and evaluation concerning implementation and dissemination of IBHC is a growing area of focus [104]. In a systematic review, Oxman, Dietrich, and Schulberg [11] explained that studies on IBHC in the early 2000s had increased focus on translation, dissemination and sustainability, especially concerning system and practice redesign. Practice culture supporting change and established buy-in for IBHC are important precursors to implementation. Ultimately, primary care providers do value care management, mental health integration, and education [105],[106], but it can take time to reach this realization. At the organizational or administrative level, leadership must recognize the inherent challenges associated with change, and take care to engage practices in and adequately prepare them for the change process. In an adequately prepared practice, primary care clinicians and mental health specialists have received training in enhanced “consultation-liaison skills” and following new practice protocols, and have been engaged in planning [24].

Engaging stakeholders in planning and implementation is central to the Depression in Primary Care program (supported by the Robert Wood Johnson Foundation), which encourages engagement of six groups of stakeholders – 1) patients/consumers, 2) providers, 3) practice/delivery systems, 4) plans, 5) purchasers, and 6) populations/policies [107]. Another unique focus to this framework is the inclusion of economic considerations and innovative financial incentive arrangements, and the encouragement of collaborations between care providers and payers. The mental health integration program at Intermountain (one of the Depression in Primary Care grantees) was evaluated in terms of patient and provider satisfaction, patient and family health, functioning and productivity, and cost neutrality, using cohort and cost-trend analysis to show changes over time in outcomes in the system [84]. In a quasi-experimental, retrospective cohort study comparing 73 out of 130 clinics that had implemented the mental health integration program with those that had not, patients in the treatment cohort had a lower rate of increase in costs than those in usual care—especially for those with depression and at least one other comorbidity [108].

The Veteran Health Administration (VHA) Quality Enhancement Research Initiative (QUERI) is a methodology for quality improvement and mixed methods evaluation of implementation and dissemination of evidence-based practices [109]. QUERI is the evaluation framework for national implementation and dissemination of IBHC in the Translating Initiatives for Depression into Effective Solutions (TIDES) efforts [110],[111]. In TIDES, the importance of the national leadership, sustainable business models, and clinical feasibility and effectiveness is explicit. There is an emphasis on determining elements of IBHC that should be standardized vs customized across the different sites (i.e., the extent to which there should fidelity vs flexibility in the model). There is evidence that translation of the TIDES model into practice leads to better depression outcomes; they have also seen increased support for the TIDES model at the national policy level [112].

The “Depression Improvement Across Minnesota, Offering a New Direction” (DIAMOND) project was a large scale implementation of IMPACT throughout the state of Minnesota, using a new payment mechanism agreed upon by participating payers. Six components of IBHC were implemented in DIAMOND: depression screening using the PHQ-9, tracking and monitoring with a patient registry, stepped care for depression, relapse prevention planning, care management, psychiatric consultation and supervision. The DIAMOND evaluation was a staggered implementation, multiple baseline design based on methods for practical clinical trials, with a focus on translation and dissemination outcomes [113]. Among the benefits of this approach are increased generalizability to diverse patient populations and practice settings, as well the potential to evaluate reach and organizational context [113].

Several publications on DIAMOND have focused on implementation. The “implementation chain” for DIAMOND starts with organization priority setting and reimbursement change, then developing change processes and practice systems (supported by training and facilitation resources), ultimately leading to improved outcomes at the patient level [114]. Notably, results from DIAMOND practice surveys show that practices are more successful at implementing collaborative care for depression when provided with financial support, training and facilitation, and specific mental models, care processes, workers and expertise [114]. Other publications report on relationships between how practices implemented the model and patient outcomes. Strong leadership support, well-defined care manager roles, a PCP champion, and an on-site and accessible care manager were associated with reach (patients entering the program), while having an engaged psychiatrist, not perceiving operating costs as a barrier, and face-to-face communication between care managers and PCPs were associated with effectiveness (patients symptom-free at 6 months) [115]. A stepped-wedge evaluation of DIAMOND was just published, showing increased receipt of depression services and greater patient satisfaction among DIAMOND care patients than comparison group patients, but no difference in depression remission rates [116].

## 9. Costs and Sustainability

Finally, while the evidence on paying for and sustaining IBHC has not yet caught up with the changes in reimbursement and financial models supported by the ACA, there is a literature on costs associated with IBHC. The sustainability of integrated care models can be tenuous [117], especially in resource-limited safety net settings [118]. The high cost of these programs, in terms of workforce, information technology, time and space, is an obvious barrier to sustainability, often representing the primary resistance to implementing IBHC [106]. Many of these programs are supported by temporary grant funding and foundation support, or are implemented in resource-rich health maintenance organizations. Many evaluations of financial outcomes have followed reports of clinical outcomes for a range of study designs, from randomized trials to program evaluation, in the context of providing behavioral health services in medical settings [119]. Generally speaking, integrated mental health care is more acutely expensive than usual care, but yields better outcomes and may offset costs in the long run [117], especially when considering the positive impact on healthcare utilization and productivity [13]. Payment models that enable billing and payment for integrated mental health services are needed [72],[119]. Currently, initiatives are underway to test mechanisms that allow for better financial support of IBHC for large scale adoption and scalability.

One such initiative is SHAPE: Sustaining Healthcare Across integrated Primary care Effort, a 3 year evaluation of a global budget for integrated services. The sample includes six primary care practices on the Western Slope of Colorado and has three main objectives: 1)to determine if a global payment method will financially support and sustain behavioral health in primary care; 2) to understand how different payment models will affect clinical models of integration and their related costs; and 3) to test the real world application of a global methodology on primary care practices who have integrated behavioral health with the end goal to inform policy. The global payment model includes risk adjusted prospective payment for defined period, shared risk & accountability in budget and quality targets between practices and payer, and incentive opportunity for quality improvement in patient health outcomes. Results of this initiative are forthcoming [120].

***Reimbursement.*** Healthcare providers are accountable for the care they deliver and payment models reward the delivery of effective care and good patient outcomes. A payment model associated with improved patient outcomes is the Washington state Mental Health Integration Program [121]. Patient registries that incorporate IBHC can aggregate data on clinical processes and patient outcomes and can be used as a basis for compensating providers not only for the quantity but also for the quality and outcomes of services provided. Payers have also displayed interest in mHealth, and the economic pressure for more patient-centered, preventive care is likely to drive them further towards the patient's viewpoint [90]. For example, there are two CPT codes (99090: collect and review data from patient; and 99091: computer data analysis) for collection of patient-generated data and review of results with the patient, which yields about $56 a month from CMS [89]. There is also opportunity for reimbursement innovation associated with tracking devices in chronic disease cases where the potential is high for reducing reimbursement [89].

The Health Information Technology for Economic and Clinical Health (HITECH) Act invests $26 billion in HIT by providing financial incentives for adoption of EHRs. This legislation seeks to promote the spread of EHRs to improve healthcare, with implementation. Medicare and Medicaid provide incentives coupled with penalties beginning in this year for providers who fail to adopt EHRs that meet meaningful use standards [122]. The inclusion of patients as HIT users creates a need for well-integrated patient-facing and provider-facing tools intended to support patient empowerment, care coordination, improved patient outcomes, population-based care, and quality-improvement [123],[124].

These goals share substantial overlap with the principles and activities characteristic of effective IBHPC and demonstrate the need to pair HIT with appropriate service delivery models. A significant gap remains in our understanding of how to sustain the integrated care interventions demonstrated in these relatively richly resourced environments in small to medium sized, independent primary care practices. This is where the ACA can be most beneficial – creating opportunities to pay for behavioral health specialists and care managers. Emerging models of pay for performance and accountable care organizations (ACOs) are dramatically restructuring the incentives for chronic disease care delivery, and may serve as a boon for attempts to implement IBHC sustainably. In addition, further research is needed at multiple levels within various systems to demonstrate the impact of IBHC on cost savings to determine which interventions or models of care result in reduced emergency room visits and hospitalizations, reduced need over time for specialty medical care, improved efficiency within the clinical setting, and overall improved population health in areas of mental health, health behavior change, and substance abuse and dependence. Other ways of demonstrating the value of IBHC has to do with examinations of improved job satisfaction and decreased burnout within primary care teams [125].

## 10. Summary

In summary, there is strong evidence supporting IBHC, and meta-analyses show that IBHC can lead to better outcomes for many patients – especially those with depression. A majority of studies have focused on outcomes in patients with a mental health diagnosis, and on IBHC approaches involving care management for depression. However, this is quickly changing, and evidence for other IBHC models for a variety of behavioral health concerns is emerging. Screening and treatment for depression and substance abuse have received the most rigorous evaluation through randomized controlled trials, and are thus backed by the strongest empirical support. Common elements across studies include standardized screening standards for identification of patients; clinical care management services and consistent follow-up; medical monitoring and medications; and brief behavioral health treatment within the primary care setting. With new evidence emerging of the equivalent efficacy of behavioral treatments to medication, availability of IBHC is likely to gain importance [126]. Variations include the type, level of training and expertise of behavioral health specialists to be employed, the populations targeted, clinical processes, degree of collaboration and reliance upon face-to-face vs remote/asynchronous communication amongst the care team. Barriers to implementation include financial factors, workforce shortages, limited HIT systems, challenges inherent to practice change, and communicating the value of IBHC to providers and patients. Those seeking to implement IBHC have many decisions to make concerning the care team, practice preparation, populations targeted, the setting, HIT, and strategies for implementation and sustainability.

More evidence is needed for payment models and practice improvement in IBHC, training and workforce development, and on strategies for implementation and large scale dissemination in real-world settings with complex patient populations. Classic randomized trial designs may not fit with measuring such dynamic and complex interventions, which has resulted in narrowly focused research on the establishment of only small numbers of short-lived programs that show minimal impact on healthcare policy and funding. The evidence-based foundation for IBHC is hindered by narrow definitions of quality empirical research. New research paradigms are needed to accommodate comprehensive and complex primary care questions about essential elements, effective interventions, and implementation strategies for IBHC. Innovative techniques such as mixed quantitative and qualitative methods, pragmatic trials and other process-observational approaches have the potential to define more comprehensively what constitutes behavioral healthcare, translate key components of IBHC within local healthcare settings, and determine the effectiveness of such approaches.

The ACA has great potential to promote a global vision of offering whole person-centered care and provide opportunity for integrated behavioral health care to become embedded in primary care. It is time for the behavioral health field to not only advocate for change within the system, but to redefine itself as a central player in healthcare as a whole, rather than a distinct and separate subspecialty.
